# Mg Doping of N-Polar, In-Rich InAlN

**DOI:** 10.3390/ma16062250

**Published:** 2023-03-10

**Authors:** Ján Kuzmík, Ondrej Pohorelec, Stanislav Hasenöhrl, Michal Blaho, Roman Stoklas, Edmund Dobročka, Alica Rosová, Michal Kučera, Filip Gucmann, Dagmar Gregušová, Marian Precner, Andrej Vincze

**Affiliations:** 1 Institute of Electrical Engineering, Slovak Academy of Sciences, 831 04 Bratislava, Slovakia; 2 International Laser Centre, Slovak Centre of Scientific and Technical Information, 811 04 Bratislava, Slovakia

**Keywords:** GaN, InAlN, acceptor doping, electron mobility

## Abstract

Metal organic chemical vapor deposition was used to grow N-polar In_0.63_Al_0.37_N on sapphire substrates. P-doping was provided by a precursor flow of Cp_2_Mg between 0 and 130 nmol/min, reaching a Cp_2_Mg/III ratio of 8.3 × 10^−3^. The grain structure of 360 nm thick InAlN was spoiled by pits after introducing a flow of CP_2_Mg at 30 nmol/min. The surface quality was improved with a flow of 80 nmol/min; however, detrimental deterioration appeared at 130 nmol/min. This correlated with the XRD shape and determined density of dislocations, indicating a phase separation at the highest flow. Degenerated n-type conduction and a free carrier concentration of ~10^19^ cm^−3^ were determined in all samples, with a minor compensation observed at a CP_2_Mg flow of 30 nmol/min. The room temperature (RT) electron mobility of ~40 cm^2^/Vs of the undoped sample was reduced to ~6 and ~0.3 cm^2^/Vs with a CP_2_Mg flow of 30 and 80 nmol/min, respectively. Scattering at ionized acceptor/donor Mg-related levels is suggested. RT photoluminescence showed a red shift of 0.22 eV from the virgin 1.73 eV peak value with Mg doping. Mobility degradation was found to be the main factor by InAlN resistivity determination, which increased by two orders of magnitude, approaching ~0.5 Ωcm, at the highest Cp_2_Mg flow.

## 1. Introduction

There is a need for novel material systems that can open a window for sub-THz-frequency electronics. In this respect, because of its record calculated electron velocity, InN is among the hottest candidates [1]. Indeed, an evaluation of the state-of-the art InN, which was grown by molecular-beam epitaxy, indicated an electron velocity of approximately 1 × 10^8^ cms^−1^ [2]. However, an experimental demonstration of InN-channel transistors is missing to date because of the large misfit between InN and ordinary GaN buffers. Elsewhere, it was suggested that InAlN buffer layers with a high In content could resolve this issue by providing better matching [3]. Additionally, if an InAlN buffer is grown with N-polarity, the InN channel layer can stay on the top of the epitaxial structure [3]. 

The In_0.6_Al_0.4_N reported by us was grown at approximately 720 °C by using metal-organic chemical vapor deposition (MOCVD) [4,5]. By employing a short nitridation and direct growth on a sapphire substrate, N-polarity and a high In content in a readily relaxed InAlN layer were obtained. The orientation of the bonds was explained by a dissociation of an AlON interlayer and verified using high-resolution transmission electron microscopy [4,5]. Occasional inversion domains were linked with the formation of isolated Al-polar AlN islands [5]. Elsewhere, an off-axis sapphire cut was suggested to suppress these irregularities [6,7]. Finally we grew on-axis N-polar InN/InAlN heterostructures, which were unique in having an InN channel (transistor-like) thickness of only 20 nm. Still, the room temperature (RT) electron mobility was as high as 720 cm^2^ V^−1^ s^−1^ [7]. Reduction of the InN dislocation density by diminishing the still-existing misfit to InAlN was suggested to enhance the electron mobility further [7]. It was also emphasized that for the functionality of InN-channel transistors, the compensation of unintentional donors in the InAlN buffer layer is necessary.

Unfortunately, In-rich InAlN compounds show inherently degenerate n-type conduction as the Fermi level rises above the conduction band with an In fraction higher than ~0.6 [8]. This was reflected by the Hall-method-determined free electron concentration of ~10^19^ cm^3^ in an undoped, MOCVD-grown In_0.57_Al_0.43_N reported by us [7], or in InN even after p-type doping [9]. Still, the compensation of residual donors in III-N buffer layers using relatively easily ionized Mg acceptors (when compared with C or Fe acceptors) may be the most viable method, as was already demonstrated in AlGaN/GaN transistors [10]. In MOCVD, Mg-doping is typically achieved by adding the bis(cyclopentadienyl) magnesium Cp_2_Mg precursor flow [9,10,11,12,13]. In GaN layers, when a Cp_2_Mg/trimethylgalium (TMG) flow ratio between 0.6 and 1.6 × 10^−3^ is used, the Mg concentration reaches approximately 2 × 10^19^ cm^−3^ [11,12]. In such cases, as the Mg-related acceptor level is 0.25 eV above the valence band, providing an ionization rate of approximately 4 % at RT, the hole concentrations are ~10^17^ cm^−3^ [10,11,12]. In other words, GaN can easily become semi-insulating or show p-type conduction if virgin GaN is only lightly n-type doped (electron concentration ≤10^17^ cm^−3^). However, for Cp_2_Mg/TMG ≥ 2 × 10^−3^, Mg becomes amphoteric, while the GaN crystallographic quality deteriorates with an appearance of pyramidal defects and Mg segregation [11,12]. The situation is slightly different for InGaN compounds in which, similar to InAlN, the concentration of residual free electrons increases with the In molar fraction, which requires a more intense delivery of Mg to achieve full compensation and/or p-type conduction [13]. In particular, Cp_2_Mg/(triethylgalium (TEG)+trimethylindium (TMI)) as high as ~4 × 10^−2^ was needed to induce holes in In_0.2_Ga_0.8_N while maintaining decent crystallographic quality. On the other hand, phase separation occurred for the same flows but a higher In molar fraction of 0.37 without achieving p-type conduction [13].

As indicated above, the complete compensation of donors and/or hole (p-type) conduction in In-rich/In-containing compounds is hard to achieve. In particular, MOCVD-grown InN showed only n-type conduction in the whole range of Mg doping, with only a slight drop in conduction at the lowest Cp_2_Mg/TMI flow ratio of 5 × 10^−3^ [9]. In InN reported elsewhere, p-type conduction was suggested. This claim however, was based only on a capacitance–voltage analysis without performing the Hall experiment [14]. As high-background n-type doping demands a high Mg concentration, problems arise due to Mg’s amphoteric behavior and increase in dislocations. With a high density of dislocations, free carrier mobility also becomes severely limited [15].

Mg doping also significantly influences the optical properties of III-N layers [9,10]. The low-temperature photoluminiscence (PL) spectra of GaN showed several Mg-related peaks within the bandgap with a rising intensity depending on the Cp_2_Mg flow. The peaks merged into a single broad signal for an Mg concentration of 8 × 10^18^ cm^−3^ when the virgin bound exciton at 3.48 eV practically disappeared [10]. On the other hand, the RT PL of optimized p-type N-polar GaN was dominated by a single donor–acceptor peak separated by ~0.12 eV from the near band-edge emission at 3.4 eV [16]. With increasing Mg doping, an additional blue-luminescence peak at 2.8 eV was assigned to self-compensation [16].

In our work, we studied the Mg doping of MOCVD-grown, N-polar InAlN with an In molar fraction of approximately 0.62. The Cp_2_Mg/(trimethylaluminum (TMA) + TMI) ratio was increased to a level of 8.3 × 10^−3^ at which the material resistance began to saturate and InAlN phase separation occurred. 

## 2. Experiment

The InAlN growth was performed using an AIXTRON 3 × 2″ flip-top, close-coupled showerhead MOCVD system; the process recipe was analogous to our previous reports [4,5]. Initially, c-plane sapphire substrates were exposed to NH_3_ at approximately 1015 °C for 300 s. After ramping down to 710 °C, the AlN interlayer growth was performed for 35 s. In respect of the InAlN growth, TMA, TMI, NH_3_, and the total flow were kept at 4.74 µmol/min, 10.95 µmol/min, 3 slm, and 10 slm, respectively, targeting an In molar fraction of 0.63. The Mg doping was varied by setting the Cp_2_Mg flow between 0 and 130 nmol/min, reaching the highest CP_2_Mg/(TMA + TMI) flow ratio of 8.3 × 10^−3^. The InAlN growth was performed at the pressure of 20 kPa for 8440 s. The growth process was monitored using an in situ LayTec EpiCurve^®^TT optical (632.7 nm) reflectance system.

High-resolution X-ray diffraction (HR-XRD) characterization was performed using a Bruker D8 DISCOVER diffractometer with a rotating Cu anode operating at 12 kW. InAlN diffractions $\left(0004\right)$ and $\left(11\bar{2}4\right)$ diffractions were measured to determine the molar fractions, lattice parameters, and strain states [7]. The density of the screw- (N_disS_) and edge-type dislocations (N_disE_) were evaluated from the symmetric—$\left(0002\right)$ and skew—$\left(10\bar{1}1\right)$ diffractions. Calculations were performed assuming N_disS_ = $\frac{{Г}_{0{}^{\circ}}^{2}}{1.88b_{s}^{2}}\;,$ N_disE_ $=\frac{{Г}_{90{}^{\circ}}^{2}}{1.88b_{e}^{2}}\;,$ where ${Г}_{\chi }$ represents the full-width at half-maximum (FWHM) of the rocking curves at the inclination angles χ=0° and 90°, and b_s_ = c and b_e_ = a are the Burgers vectors of the corresponding dislocations [17,18].

The depth-resolved elemental composition of the Mg-doped InAlN was analyzed using secondary ion mass spectroscopy (SIMS) by Ion-TOF GmbH. An NT-MDT NTEGRA Prima atomic force microscope (AFM) in tapping mode and a FEI Quanta 250 FEG scanning electron microscope (SEM) were used to study the surface morphology of the grown samples. The TEM analyses were performed by conventional TEM JEOL JEM1200EX, using cross-sectional TEM specimens prepared with the FIB technique.

The Hall transport parameters were measured in the temperature range between RT and 4 K using a standard Van der Pauw configuration. The resistivity of the grown layers was also investigated using transmission-line-method (TLM) test structures [19], with differently spaced, non-annealed ohmic contacts composed of Ti/Al/Ni/Au. RT PL spectra were acquired using an Ar^+^ laser at 488 nm for sample excitation and an Si photodiode for signal detection.

## 3. Results and Discussions

**Figure 1:**
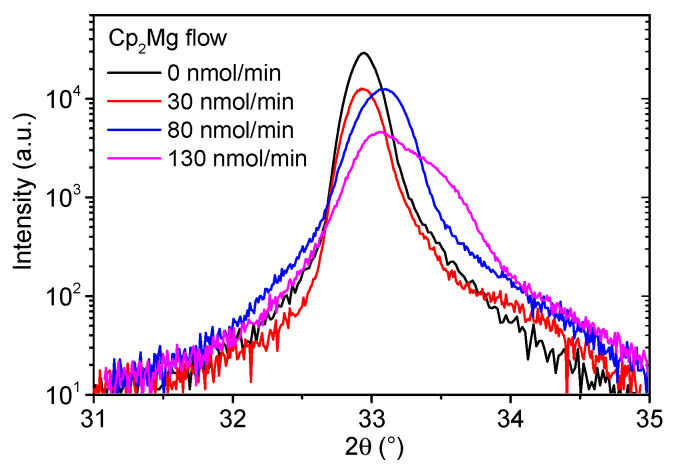
XRD 2 Θ/ω scan of InAlN (0002) XRD diffractions for different Cp_2_Mg flow.

The XRD analysis of InAlN (0002) diffractions shown in Figure 1 indicates that by increasing the CP_2_Mg flow to 80 nmol/min, the position of the (0002) reflection shifted to higher angles while the FWHM of the signal increased. This was caused by a minor decrease in the In molar fraction (by up to 0.03) and by generating additional screw- and edge-type dislocations (a combined increase of 45 % for the CP_2_Mg/(TMA + TMI) flow ratio of 8.3 × 10^−3^), quantified in Table 1. An allusive side maximum was notable for the highest CP_2_Mg flow, suggesting that the InAlN underwent phase separation. A minor reduction in the growth rate with the introduction of the Mg doping was also observed.

**Figure 2:**
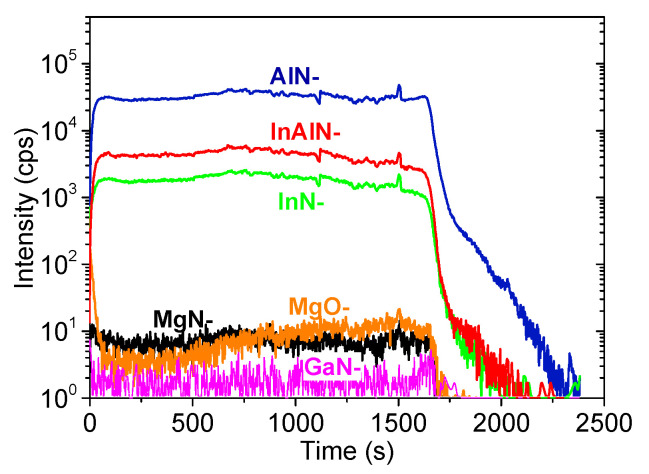
SIMS depth analysis of InAlN grown with 130 nmol/min Cp_2_Mg flow. X-axis refers to sputtering time by profiling.

Figure 2 shows the elemental SIMS profiles of the InAlN sample doped at a 130 nmol/min CP_2_Mg flow. MgO^−^ and MgN^−^ ions were detected with an almost stable intensity of ~10 counts/s in good alignment with those of the other InAlN elements. This proved a stable concentration of Mg through the sample cross-section with a marginal memory effect at the onset of doping. We note that unless a careful calibration of the SIMS method in In_0.6_Al_0.4_N is performed, any Mg quantification is meaningless. Nevertheless, taking into account the relatively high CP_2_Mg/(TMA + TMI) flow ratio when compared to the CP_2_Mg/TMG ratio of GaN doping [11,12], we expect that the Mg concentration might be in the range of 10^19^–10^20^ cm^−3^.

**Figure 3:**
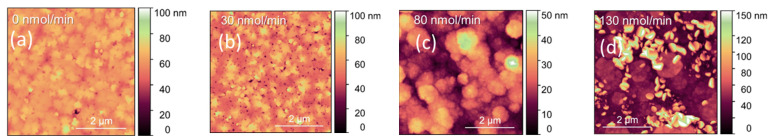
InAlN layers AFM analysis for Cp_2_Mg flow of (**a**) 0 nmol/min, (**b**) 30 nmol/min, (**c**) 80 nmol/min, and (**d**) 130 nmol/min.

**Figure 4:**
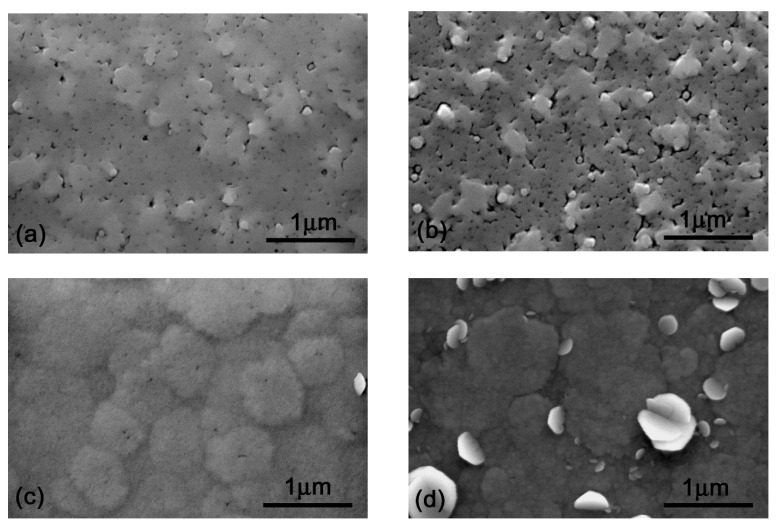
InAlN layers SEM analysis for Cp_2_Mg flow of (**a**) 0 nmol/min, (**b**) 30 nmol/min, (**c**) 80 nmol/min, and (**d**) 130 nmol/min.

A typical grain mosaic structure [7,20] and a root mean square (RMS) roughness of 5.9 nm were deduced from the 5 × 5 μm^2^ AFM scan of the undoped InAlN, shown in Figure 3a. A number of pits and surface roughness deterioration (RMS ~8.9 nm) appeared with the introduction of CP_2_Mg flow of 30 nmol/min, shown in Figure 3b. Point defects disappeared as the flow was further increased to 80 nmol/min in addition to the enlargement of grains and smoothing of the InAlN surface (RMS ~7.5 nm), shown in Figure 3c. Interestingly, these surface improvements at a CP_2_Mg flow of 80 nmol/min correlated well with a decreased density of screw-type dislocations, shown in Table 1. Finally, the surface detrimentally deteriorated and exhibited a number of misoriented grains at a CP_2_Mg flow of 130 nmol/min (Figure 3d). This partially surprising trend (smoothing of the surface with a CP_2_Mg flow of 80 nmol/min) is also well illustrated in the SEM views shown in Figure 4.

**Figure 5:**
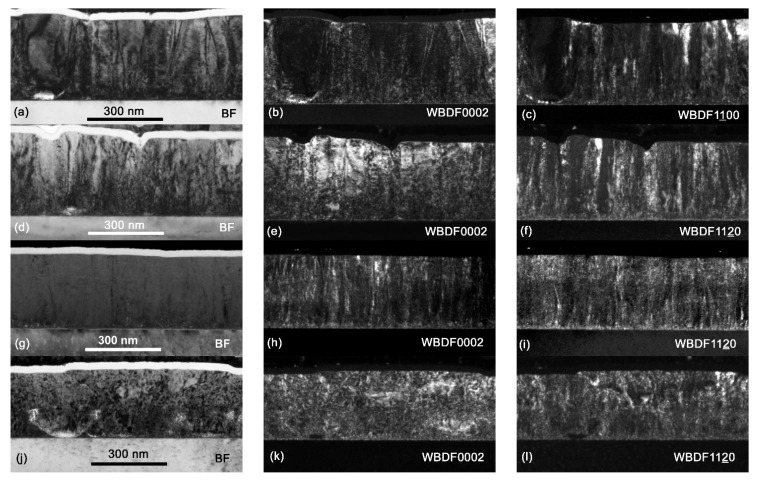
InAlN layers TEM analysis for Cp_2_Mg flow of (**a**–**c**) 0 nmol/min, (**d**–**f**) 30 nmol/min, (**g**–**i**) 80 nmol/min, (**j**–**l**) 130 nmol/min. The images are taken as bright field (BF) or weak beam dark field (WBDF) images using diffraction beams indicated in the images.

A TEM analysis revealed inner microstructure changes due to different Cp_2_Mg flows during the layer growth. Figure 5 presents bright field (BF) and weak-beam, dark-field (WBDF) images using the specimen orientations specific for the visualization of dislocations. The weak-beam, dark-field image using 0002 diffraction illustrates a situation in which the invisibility criterion is fulfilled for pure edge dislocations, while the WBDF using $1\bar{1}00$ or $11\bar{2}0$ diffractions makes pure screw dislocations invisible. The TEM analysis becomes important for defects which cannot be easily reflected by an XRD analysis of InAlN 0002 diffractions. In particular, grains with a larger, out-of-plane rotation are shown as U-shaped grains in Figure 5a–c for the undoped InAlN. The addition of a Cp_2_Mg flow of 30 nmol/min did not suppress the U-shaped grains (as was indicated by SEM in Figure 4b) and increased the surface roughness. However, when out-of-plane grain growth was suppressed, the InAlN layer surface smoothness was improved, and the majority of pits also disappeared in the layer grown with a Cp_2_Mg flow of 80 nmol/min (Figure 4c and Figure 5g–i for SEM and TEM, respectively). Simultaneously, stacking faults were introduced into the sample and are reflected as the layered structure shown in Figure 5g–i. In general, by summarizing results from the XRD, AFM, SEM, and TEM analyses, we can conclude that by introducing a sufficiently high Cp_2_Mg flow, the microstructure of InAlN layers was improved with fewer pits and a suppressed growth of misoriented grains. However, the last sample with the highest Cp_2_Mg flow showed a high level of lattice disorder (Figure 5j–l), connected with a loss of the continuous “columnar” growth and an initiated, intense growth of conical grains with a large, out-of-plane misorientation (shown in Figure 4d).

**Figure 6:**
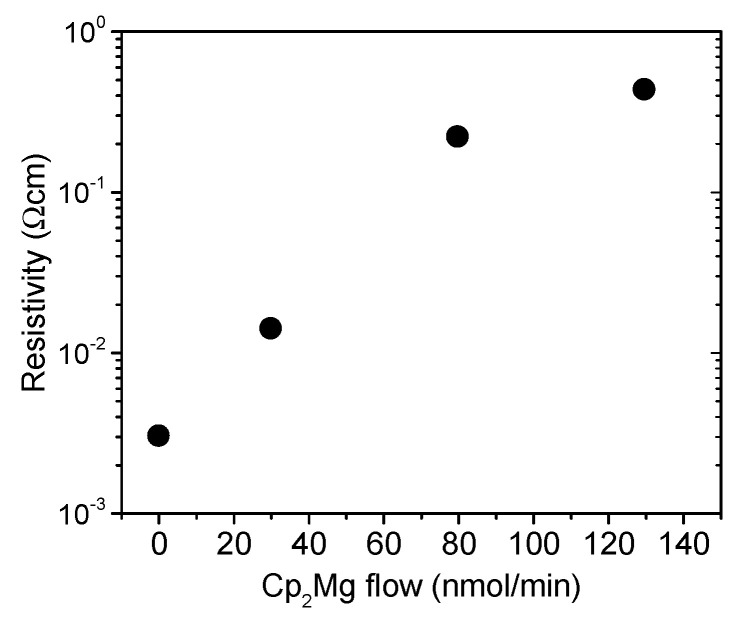
InAlN resistivity TLM method values for different Cp_2_Mg flow.

Our findings can be further correlated with the InAlN resistivity values extracted using the TLM method, shown in Figure 6. When compared with the undoped InAlN, Mg doping led to a steep resistivity increase by two orders of magnitude, reaching ~0.2 Ωcm for the CP_2_Mg flow of 80 nmol/min. On the other hand, a tendency of resistivity saturation could be observed at the flow of 130 nmol/min.

**Figure 7:**
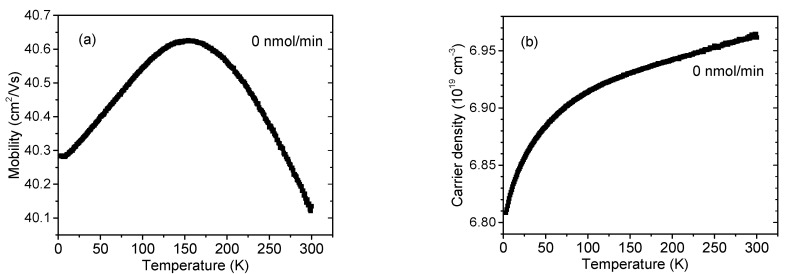
InAlN layer free electron (**a**) mobility and (**b**) concentration. Cp_2_Mg flow during the growth was 0 nmol/min.

As was pointed out in the Introduction, the resistivity increase observed by the TLM measurement is an expected and desired outcome of the InAlN Mg doping. To analyze the electric conduction further, we separated the free carrier mobility (*μ*) and concentration (*N*) quantities using Hall experiments from RT down to 4 K. This analysis was performed for all samples except for the one prepared at the 130 nmol/min flow, as that sample was already structurally degraded. In the following, we used the CP_2_Mg flow rate as a label to distinguish the *μ* and *N* data. What we found in all cases was an n-type conduction, even though various *T*-dependences were observed. For the undoped InAlN (CP_2_Mg flow = 0) we acquired a bell-like *T*-dependence for *μ*(0), shown in Figure 7a, and a partial freeze-out for *N*(0), shown in Figure 7b. This implies a combination of scattering at ionized impurities, particularly at the low temperature range and at charged threading dislocations [15,21]. However, *μ*(0) changed only slightly, and thus both the scattering mechanisms with opposite *T*-dependence seem to be almost balanced, as similarly observed elsewhere for highly doped and defective GaN [15]. Regarding the scattering centers, in the undoped InAlN, similar to GaN, unintentional impurities can be linked to N vacancies and/or oxygen atoms [16] which are responsible for the high background electron concentration. Electrons can be partially trapped at their respective donor states as *T* decreases, shown in Figure 7b. High concentrations of threading dislocations are typical for the In-rich InAlN [20], and the same was observed in our case, shown in Table 1.

**Figure 8:**
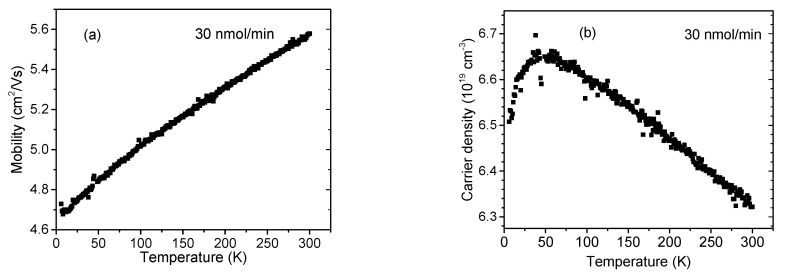
InAlN layer free electron (**a**) mobility and (**b**) concentration. Cp_2_Mg flow during the growth was 30 nmol/min.

With adding a CP_2_Mg flow of 30 nmol/min, the situation changed significantly. In this case, the mobility dropped by one order of magnitude compared with that of the undoped InAlN, while *μ*(30) increased steadily with *T,* shown in Figure 8a. This scenario strongly indicates the dominance of a new impurity scattering mechanism related to the Mg doping. We do not assume a significant role of threading dislocations in this case as their density remained almost intact. As was reported earlier for GaN, Mg doping generates various new levels and complexes, such as Mg_Ga_ or multiple-charged (Mg_Ga_ –V_N_) [21]. Similar Mg-related levels can be expected in our study, representing new impurity scattering centers. We noticed an approximately 10 % lower *N*(30) compared to *N*(0), likely because of the partial compensation of donors, shown in Figure 8b. However, compared with the undoped InAlN, *N*(30) decreased with an increasing *T* between ~50 and 300 K. The reason for this is unknown to us. We can speculate that some kind of phonon-assisted compensation mechanism took place, similar to what was reported for the (Mg_Ga_–V_N_)-related phonon-assisted luminescence transitions in GaN [21].

**Figure 9:**
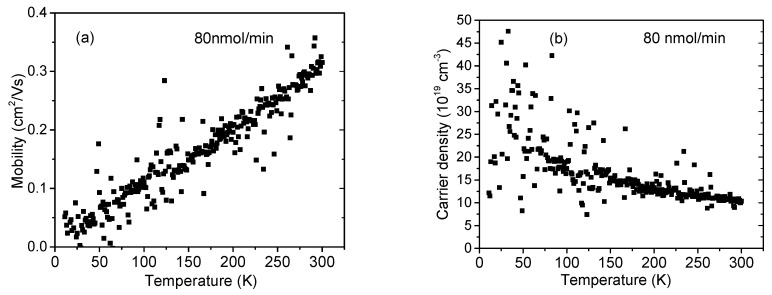
InAlN layer (**a**) electron mobility and (**b**) free electron carrier concentration. Cp_2_Mg flow during the growth was 80 nmol/min.

**Figure 10:**
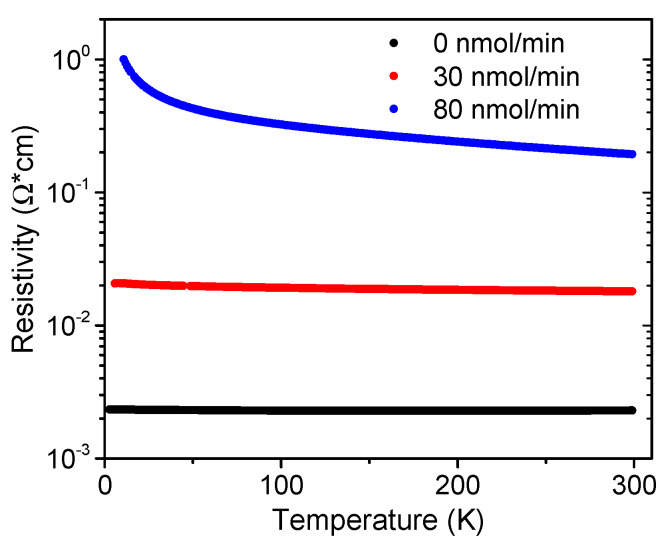
Dependence of the InAlN Hall resistivity on temperature for different Cp_2_Mg flows.

Finally, the observed trend in *N* and *μ*with the CP_2_Mg flow became even more pronounced for 80 nmol/min, shown in Figure 9. In this case, however, *N*(80) was higher than *N*(0), suggesting amphoteric behavior of Mg. Still, *μ*(80) exhibited an additional dramatic decrease, likely because of new, Mg-related ionized impurities and/or defects in InAlN. Consequently, the flow of 80 nmol/min produced the most resistive sample (see the evolution in Figure 10). The acquired data in Figure 9 are scattered because the highly resistive samples were measured with insufficient precision.

**Figure 11:**
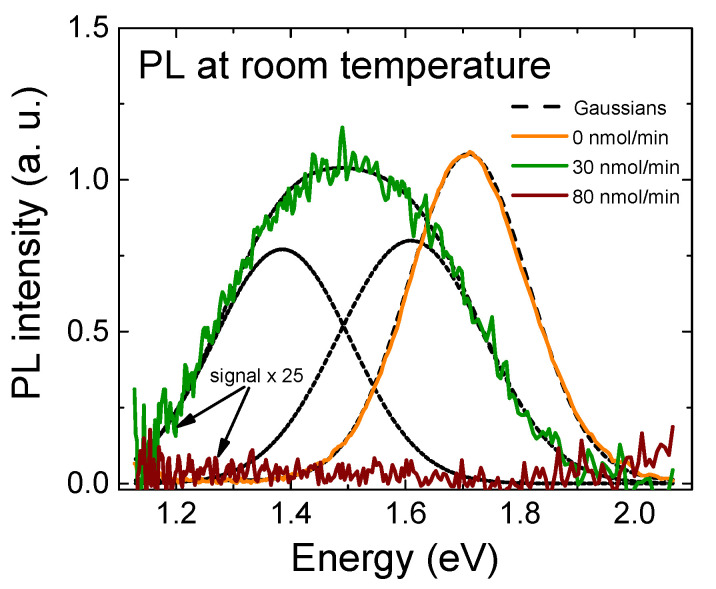
InAlN room temperature photoluminescence for different Cp_2_Mg flow. Combination of two Gaussian curves fit the 30 nmol/min sample.

In Figure 11, we compare the RT PL signal of the undoped InAlN sample with the two p-doped samples grown at CP_2_Mg flows of 30 and 80 nmol/min. The PL maximum of the undoped InAlN appeared at ~1.71 eV and was well correlated with the XRD-detected In molar fraction of 0.62 and in agreement with expected bowing of the InAlN band gap dependence [8]. With the introduction of Mg doping, the peak of the PL signal was red-shifted by ~0.22 eV, similar to what was reported for the donor–acceptor pair in Mg-doped GaN [10]. Our finding is in agreement with the Hall experiment, shown in Figure 8b, where the partial compensation of residual donors was linked to Mg-related acceptors. We also noticed an increase of the PL FWHM from 258 meV to 466 meV. Finally, RT PL was also taken from the InAlN doped at the higher CP_2_Mg flow of 80 nmol/min. In this case, however, the signal dropped below the detection limit. This can be explained by the Mg’s amphoteric behavior, which provided non-radiative recombination through newly generated donor levels or defects, similar to what was reported for highly Mg-doped InN [9].

The PL maximum shift introduced by doping could be a consequence of the radiative recombination involving acceptors and of the effective band gap narrowing caused by defect-related potential fluctuations. In the semiconductor with a high doping level, the fluctuations stemmed from a random dopant distribution in combination with some crystalline defects. Their spectra typically reflect the Gaussian distribution of probability. In a ternary compound, they also stem from local fluctuations of the composition. A basic formulation of the band-tail-state model was created by Eliseev et al. [22,23], and it was then developed into the PL model of the localized state ensemble by Li et al. [24]. The model was used recently by Hidouri et al. to quantitatively evaluate the temperature dependence of PL band features in ternary and quaternary epitaxial layers [25,26,27] and the respective quantum wells [28].

To estimate the extent to which the density of states in our samples correspond to the tail-state model, we made Gaussian fits to the PL spectra (shown in Figure 11). The PL spectrum of the undoped sample could be almost ideally fitted by a single Gaussian line. This is a sign that the dominant part of the transitions was most probably a recombination through the band-tails. For the 30 nmol/min Mg-doped sample, the spectrum could be described by a combination of two Gaussian curves shifted in the energy levels (with the maxima at 1.385 eV and 1.610 eV, respectively). One could speculate whether this is the result of a mix of band-to-band and band–acceptor transitions, both shaped by the band-tail energy distribution. However, this should be further proven by a broader and more representative set of experiments.

## 4. Conclusions

We studied the Mg doping of MOCVD-grown, N-polar, In-rich InAlN with varied CP_2_Mg flows between 0 and 130 nmol/min. Degenerate n-type conduction was observed in the reference, undoped InAlN and attributed to the high In molar fraction of 0.63 and an inherent Fermi level location close to the conduction band. Scattering at ionized unintentional impurities and threading dislocations were identified as the main factors that determined the free electron mobility values from RT down to 4 K. The situation changed through the introduction of a CP_2_Mg flow at 30 nmol/min when Mg-related acceptors became the dominant scattering centers and the mobility decreased by one order of magnitude. A partial compensation of unintentional donors was also achieved when the PL signal could be interpreted considering acceptor levels ~0.22 eV above the valence band. We noticed a slight deterioration of the surface mosaic structure with the appearance of pits and a slight increase in the density of screw dislocations. However, when increasing the CP_2_Mg flow to 80 nmol/min, the surface surprisingly improved; the pits disappeared, and the mobility continued to steeply decrease. This drop can be linked to scattering on additionally generated, Mg-related levels and complexes, as the Mg’s amphoteric nature was indicated by the Hall experiment in this case. Nevertheless, with Mg doping, the InAlN resistivity increased by two orders of magnitude, which was the desired outcome of our experiment. We can suggest that the CP_2_Mg flow of 80 nmol/min was close to the upper limit of reasonable conditions. Beyond this, the InAlN underwent a phase separation and developed a number of misoriented grains at the surface, which were observed at 130 nmol/min. In general, we can conclude that by Mg doping the In-rich InAlN, the mobility of free electrons is the main factor which controls the material resistivity. In future, Mg-doped InAlN material can be tested for the buffer layer in the design of novel InN/InAlN transistor structures.

## Figures and Tables

**Table 1:** InAlN layers material parameters for different Cp_2_Mg flow. FWHM refers to 2 *Θ*/*ω* scan shown in Figure 1.

Cp_2_Mg Flow (nmol/Min)	Cp_2_Mg/ (TMA + TMI) (10^−3^)	In Molar Fraction	(0002) FWHM (°)	Density of Screw Dislocations (10^9^ cm^−2^)	Density of Edge Dislocations (10^9^ cm^−2^)	InAlN Thickness (nm)
0	0	0.63	0.23	1.7	56.7	360
30	1.9	0.62	0.25	3.8	53.4	360
80	5	0.60	0.36	3.3	70.5	330
130	8.3	0.60	0.52	4.3	80.5	300

## Data Availability

Data can be provided upon reasonable request.
